# Struggling with a Gastric Volvulus Secondary to a Type IV Hiatal Hernia

**DOI:** 10.1155/2010/257497

**Published:** 2010-10-25

**Authors:** Dafnomilis George, Pappas V. Apostolos, Panoutsopoulos Athanasios, Lagoudianakis E. Emmanuel, Koronakis E. Nikolaos, Panagiotopoulos Nikolaos, Seretis Charalampos, Karanikas George, Manouras J. Andreas

**Affiliations:** ^1^Surgical Department, Argos General Hospital, 21200 Argos, Greece; ^2^Department Internal Medicine, Argos General Hospital, 21200 Argos, Greece; ^3^Second Department of Surgery, 417 NIMTS-Nosileutiko Idrima Metohikou Tameiou Stratou (Military Veterans' Fund Hospital), Athens, Greece; ^4^1st Department of Propaedeutic Surgery, Hippokrateion Hospital, Athens Medical School, University of Athens, Q. Sofias 114 Avenue, 11527 Athens, Greece

## Abstract

Type IV hiatal hernias are characterized by herniation of the stomach along with associated viscera such as the spleen, colon, small bowel, and pancreas through the esophageal hiatus. They are relatively rare, representing only about 5%–7% of all hernias, and can be associated with severe complications. We report a 71-year-old veteran wrestler who presented to our department with a type IV paraesophageal hernia containing a gastric volvulus and treated successfully with emergency operation.

## 1. Introduction

Hiatus hernia refers to herniation of elements of the abdominal cavity through the esophageal hiatus of the diaphragm and into the mediastinum. There are four recognized types [1]. Type I is characterized by widening of the muscular hiatal tunnel and circumferential laxity of the phrenoesophageal membrane, allowing a portion of the gastric cardia to herniate upwards. Type II hernias result from a localized defect in the phrenoesophageal membrane while the gastroesophageal junction remains fixed to the preaortic fascia and the median arcuate ligament, and the gastric fundus forms the leading part of the herniation. Type III hernias are mixed types I and II, and type IV are associated with a large defect that can allow other organs, such as the colon, spleen, and pancreas to herniate. Types II, III, and IV are rare and account for, at most, 5%–15% of all hiatal hernias [2]. We report a 71-year-old veteran wrestler who presented to our department with a type IV paraesophageal hernia containing a gastric volvulus and treated successfully after undergoing emergency operation.

## 2. Case Report

**Figure 1:**
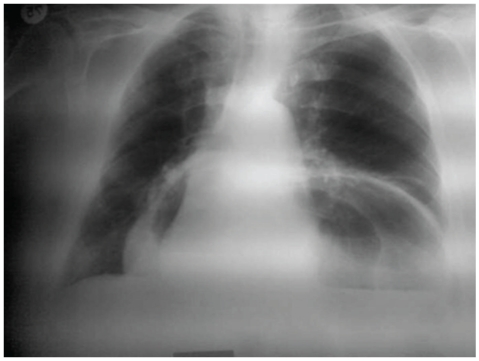
Preoperative chest roentgenogram showing a large hiatal hernia.

**Figure 2:**
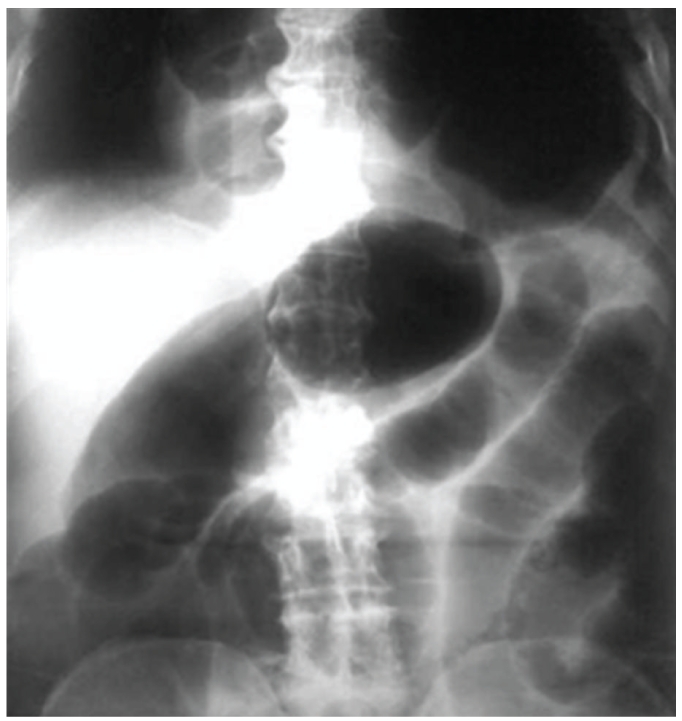
Erect abdominal X-ray reveals a large hiatal hernia with a greatly distended gastric bubble and distended bowel loops.

A 71-year-old veteran wrestler presented to the emergency department reporting progressive epigastric pain, nausea, and constipation of 3-day duration. The patient had a previous history of gastroesophageal reflux disease, for which he occasionally received antisecretory medication (H2 blockers and proton pump inhibitors). Clinical examination showed a moderately malnourished individual which was afebrile and in good general condition. Examination of the abdomen revealed generalized distension with diffuse tenderness, and chest auscultation revealed bilaterally diminished breath sounds. Laboratory tests revealed a white blood cell (WBC) count of 12.400/mm^3^ with 85% granulocytes and no other abnormalities. His chest and abdomen roentgenogram showed a large hiatal hernia (Figures 1 and 2). Considering all clinical evidence and imaging findings, gastric volvulus was suspected, and the patient was immediately led to the operating room. At laparotomy, part of the stomach, the greater omentum, and the transverse colon were found to be herniated through a markedly dilated diaphragmatic hiatus. The stomach was rotated around its long axis (organoaxial), but after a careful inspection no signs of ischemia or gangrene were found. A nasogastric tube was placed in order to decompress the stomach and allow the reduction of the hernia contents in the abdominal cavity. The hernia sac was dissected and reduced into the abdomen. The hiatal defect was closed, and a Nissen-Rossetti fundoplication was performed. The patient had an uneventful postoperative course and was discharged after 10 days. At 12 months followup, the patient has fully recovered, and a barium esophagram showed no signs of recurrence.

## 3. Discussion

Type IV hiatal hernias are characterized by herniation of the stomach along with associated viscera through the esophageal hiatus [3]. Their etiology is usually unclear; they are mainly acquired disorders resulting from the combination of an enlarged diaphragmatic hiatus with repeated episodes of elevated intraabdominal pressure [4]. Anatomical stressors, such as heavy weight lifting or even daily activities, can increase the intraabdominal pressure forcing mobile abdominal organs through the hiatus into the chest cavity [5]. Our patient was a former professional wrestler, thus explaining the long history of repeated episodes of elevated intraabdominal pressure which could predispose to hiatal hernia formation.

Paraesophageal hernias can be associated with severe complications such as intrathoracic incarceration of the stomach, bleeding, perforation, and gastric volvulus [6]. The latter is an uncommon disorder caused by the abnormal rotation of the stomach. According to the axis of rotation, it is classified into organoaxial, mesenteroaxial, and mixed type [7]. In this patient, as is in the majority of cases, the stomach was rotated around its long axis which connects the pylorus with the cardiooesophageal junction. Gastric volvulus can be primary, usually linked to the laxity of the perigastric ligaments, or more commonly secondary to para-oesophageal hiatus hernia, traumatic diaphragmatic hernia, and diaphragmatic eventration. Symptoms can be acute, such as severe pain in the upper abdomen with unproductive retching, or chronic including intermittent upper abdominal distension and dysphagia [8]. Rotation of the stomach more than 180° can result in strangulation which may lead to ischaemia, necrosis, and perforation. According to previous studies, the mortality rate can be as high as 50% in case of strangulation [9]. This high mortality rate is the result of delayed diagnosis, which increases the likelihood of catastrophic complications.

Because of the danger from these complications, emergent surgical treatment is widely recommended. Controversy exists as to the optimal technique [10]. Different methods have been proposed including open surgery and various laparoscopic techniques. According to recent studies, it appears that laparoscopic management of acute obstructed paraesophageal hernia is a safe and feasible procedure [11]. In our case open surgery was preferred based on the previous clinical experience of our department.

In summary, we presented an unusual case of gastric volvulus associated with a type IV hiatal hernia. Herniation of the abdominal contents through the diaphragmatic hiatus should be suspected in patients with vague abdominal pain and previous history of gastroesophageal reflux disease. In most cases, plain X-rays are indicative of the disease, while upper gastrointestinal series can confirm the diagnosis. As was illustrated in this case, prompt diagnosis and early surgical treatment are necessary in order to avoid any potential life-threatening complications.
